# Determination of ceruloplasmin, some other acute phase proteins, and biochemical parameters in cows with endometritis

**DOI:** 10.14202/vetworld.2016.1056-1062

**Published:** 2016-10-08

**Authors:** S. Kaya, O. Merhan, C. Kacar, A. Colak, K. Bozukluhan

**Affiliations:** 1 Department of Obstetrics and Gynecology, Faculty of Veterinary Medicine, University of Kafkas, Kars, Turkey; 2 Department of Biochemistry, Faculty of Veterinary Medicine, University of Kafkas, Kars, Turkey; 3 Department of Obstetrics and Gynecology, Faculty of Veterinary Medicine, University of Ataturk, Erzurum, Turkey; 4 Department of Veterinary Health, Kars School of Higher Vocational Education, University of Kafkas, Kars, Turkey

**Keywords:** albumin, ceruloplasmin, cow, endometritis, haptoglobin, serum amyloid A

## Abstract

**Aim:**

The aim of this study is to determine serum ceruloplasmin levels in cows with endometritis of varying degrees of severity and to establish whether or not there is a correlation between acute phase protein (APP) levels and biochemical parameters.

**Material and Methods:**

The study was conducted with 100 Brown Swiss cows (3-8 years of age) on days 28-32 postpartum. Cows were divided into endometritis (mild, moderate, and severe endometriosis) and healthy groups based on ultrasonography, vaginoscopy, and cytological examination. Blood samples were collected from all cows. Levels of haptoglobin (Hp), serum amyloid A (SAA), ceruloplasmin, albumin, and some biochemical parameters were analyzed.

**Results:**

Hp, SAA, and ceruloplasmin levels were higher in cows with endometritis than in healthy cows (p=0.001), and the levels of these APPs increased as endometritis became more severe (p=0.001). Some significant correlations were found between APPs and the biochemical parameters that were analyzed. In conclusion, it was determined that ceruloplasmin levels increase significantly in the presence of endometritis and proportionate to the severity of endometritis. A significant correlation was found between ceruloplasmin levels and Hp and SAA levels.

**Conclusion:**

It was concluded that ceruloplasmin levels can be used in the diagnosis of endometritis as an alternative to Hp and SAA levels.

## Introduction

Acute phase proteins (APPs) are blood proteins synthesized by hepatocytes as part of the innate immune system’s response to various stimuli such as inflammation, trauma, and infection [1]. The main function of APPs is to assist in defending the host against pathological damage and to restore homeostasis [2]. The levels of positive APPs (haptoglobin [Hp], serum amyloid A [SAA], ceruloplasmin, C-reactive protein, etc.) increase in response to infection, whereas the production of negative APPs (prealbumin, albumin, transferrin, etc.) is suppressed [1,3]. APPs are used in the early and accurate detection of infections in ruminants [4]. The main APPs used to detect genital tract infections and to determine treatment efficacy are Hp, SAA, and albumin [5-8]. Ceruloplasmin is a plasma α-2 glycoprotein. It is an enzyme that plays an important role in copper transport in the blood stream (95% of copper in animals) and iron metabolism (ferroxidase) [9,10]. Copper improves immune function by acting on the levels of various enzymes mediating the antioxidant system. Ceruloplasmin mediates the transport of copper by the enzymes lysyl oxidase and Cu-Zn superoxide dismutase, which play a role in tissue repair, and it plays a role in the antioxidant system. It also protects cells against oxidative damage. Phagocytosis and antimicrobial activity decrease if serum ceruloplasmin levels fall. As a result, the need for this enzyme increases in inflammatory conditions [11].

The start of lactation in the postpartum period increases the energy requirement. Body reserves are used to overcome the energy insufficiency caused by the negative energy balance [12,13]. The resulting metabolic stress causes impairment of other body functions [14]. It damages liver cells and alters some liver enzyme functions (aspartate aminotransferase [AST] and alkaline phosphatase [ALP]) [15,16]. One of the methods for detecting energy insufficiency is to determine the circulating urea concentration [17]. Although it is known that there is a significant relation between endometritis and APPs and metabolic parameters, no study investigating that the relation between the severity of endometritis and these parameters was found.

This study had two objectives. The first was to determine whether or not ceruloplasmin levels can be used as an indicator in the diagnosis of endometritis. The second was to determine changes in the levels of certain APPs and biochemical parameters according to the severity of endometritis and to establish whether or not, there is a correlation between them.

## Material and Methods

### Ethical approval

This study was conducted after obtaining approval from the Kafkas University Animal Experiments Local Ethics Committee (KAÜ HADYEK - Submission: 2016/028).

### Animals

This study was conducted on Brown Swiss cows (3-8 years of age) at the Kafkas University Veterinary School Research and Application Farm, which were fed on meadow grass, silage (live weight×1.5/100), and dairy cattle feed (20% crude protein, 2700 energy).

### Classifications and determination of endometritis

Cows that were monitored immediately after parturition (n=140) but found to have retention secundarium, vaginal tears, mastitis, and foot problems during early postpartum examinations (n=20) were excluded. Examinations to diagnose endometritis were carried out between days 28-32 postpartum. The vulva and the perineum were examined for the presence of discharge, and then a vaginoscopy was performed. The severity of endometritis in cows with discharge on the vaginal wall or the cervix uteri was recorded according to the discharge score as described by Williams *et al*. [18]. In cows that showed no evidence of discharge, the diameters of the cornua, the presence of fluid in the uterine lumen, and diameters of the cervix uteri were measured by ultrasonography (7.5 MHz, Titan^®^, Sonosite, USA). Category of endometritis was modified and classified by ultrasonographic examination which was described by Kasimanickam *et al*. [19] and Mari *et al*. [20]. Animals with an uterine discharge containing mostly translucent mucus with a small amount of pus flakes or an anechoic line in the lumen, diameter of cervix uteri <5 cm were allocated to mild endometritis (ME) group. Animals with discharge containing yellow or white flakes of pus at a percentage <50% or an anechoic fluid, mucosa with irregular edges, and diameter of cervix uteri 5-7.5 cm were allocated to moderate endometritis group (E). Animals displaying cervix and a uterine discharge containing more than 50% of yellow- and white-colored flakes of pus, occasionally sanguineous or an enlarged lumen, echogenic fluid, and diameter of cervix uteri >7.5 cm were assigned to severity endometritis (SE) group. Endometrial samples were collected from cows (n=50) that appeared to be healthy in clinical examinations using the cytobrush technique. The vulva was cleaned with a paper towel, and plastic sheaths were used to prevent contamination of the cytobrush in the vagina. Endometrial samples were obtained by rotating cytobrushes a few times on the uterine endometrium. The samples were smeared on slides and dried. They were stained with Giemsa solution (Merck^®^, Turkey). Then, the neutrophil to leukocyte ratio was calculated using microscopy (Olympus CX23, Olympus Corp, Japan) (counting a minimum of 100 cells at 400× magnification). Cows with the neutrophil to epithelial cell ratio >18% were considered to have subclinical endometritis [19]. Body condition scores (BCS) were assessed immediately after the examinations as described by Edmonson *et al*. [21] (1=thin; 5=fat; in increments of 0.25).

### Assessment of blood samples

Blood samples were collected from the vena coccygea of all animals into vacuum gel serum tubes. The samples were centrifuged at 1200 g for 10 min. The serums were then transferred to Eppendorf tubes and stored at −20°C until analysis. Hp levels were determined using the method described by Skinner *et al*. [22], and ceruloplasmin levels using the method described by Colombo and Richterich [23] (UV-1201, Shimadzu, Japan). SAA levels were determined using the ELISA kit (Tridelta Development Limited, Ireland). Albumin, AST, ALP, urea, creatinine, and total protein (TP) levels were determined colorimetrically (Epoch^®^, Biotek, USA) using the commercial test kit (DDS, Turkey).

### Statistical analysis

Statistical analyses were performed using the SPSS^®^ (SPSS 18, IL, USA) software program. Hp, SAA, ceruloplasmin, albumin, AST, ALP, urea, creatinine, and TP levels in each group were tested for normality using the Shapiro–Wilk test. Differences in BCS, age, SAA, ceruloplasmin, albumin, AST and ALP levels between the groups were compared using ANOVA and Tukey HSD tests. Hp, urea, creatinine, and TP levels were compared using the Kruskal–Wallis test, and differences were recorded. Then, the groups were subjected to the Mann–Whitney test; the correlations between Hp, SAA, ceruloplasmin, albumin, and biochemical parameters were compared using the Pearson correlation test. APP levels in cows classified according to BCS and parity were compared using the Mann–Whitney test. The results were expressed as mean±standard error, and values ≤0.05 were considered significant. Selection of cutoff points for Hp, SAA, and ceruloplasmin was undertaken using receiver operating characteristic curve (ROC) analysis. ROC analysis provides determining plots a curve of sensitivity versus specificity for all possible threshold values of the parameters. The area under the curve used to measure the diagnostic accuracy of the examined parameters [24].

## Results

### Postpartum examinations results and distribution of the groups by body condition score and age

Based on the vaginal discharge scores and the results of the ultrasonography, ME was diagnosed in 30 cows, E in 20 cows, and SE in 20 cows. 45 out of 50 cows that were examined cytologically were considered healthy. However, data from only 30 healthy cows were used in the analyses. BCS were similar in healthy cows (2.62±0.09) and cows with endometritis (ME=2.73±0.08, E=2.60±0.14, and SE=2.61±0.09), and no statistically significant difference was found (p>0.05). No significant difference was found in distribution of the groups (healthy=3.76±0.24, ME=4.03±0.16, E=4.6±0.44, and SE=3.9±0.23) by mean age (p>0.05).

### The levels of APPs (Hp, SAA, ceruloplasmin, and albumin) in groups

**Figure-1:**
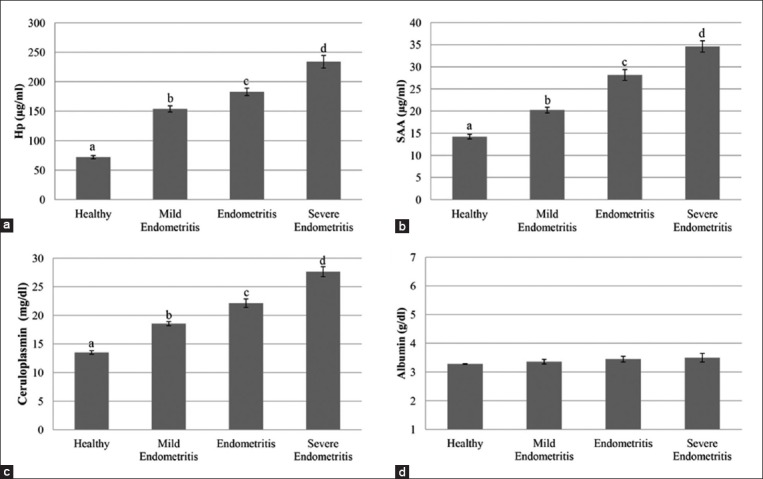
(a-d) Comparison of acute phase protein levels between groups (a:b:c:d: 0.001).

Hp, SAA, ceruloplasmin, and albumin levels in the groups are presented in Figure-1. In cows with endometritis, serum Hp (ME=154±5.16, E=183±6.2, and SE=234±10.7 µg/ml), SAA (ME=20.25±0.65, E=28.17±1.22, and SE=34.62±1.28 µg/ml), and ceruloplasmin levels (ME=18.57±0.36, E=22.14±0.75, and SE=27.64±0.87 mg/dl) were significantly higher than in healthy cows (72±2.76 µg/ml, 14.24±0.52 µg/ml, and 13.52±0.32 mg/dl, respectively) (p=0.001). Levels of albumin, which is considered a negative APP, were similar in the groups (p>0.05) (Figure-1).

Cutoff, sensitivity, and specificity values for the Hp, SAA, and ceruloplasmin levels determined as a result of the ROC analysis on healthy cows and those with endometritis (mild and moderate) are provided in Table-1. Hp, SAA, and ceruloplasmin levels did not overlap in healthy cows and those with severe endometritis. Therefore, the cutoff values were not calculated for these two groups (Table-1).

**Table-1:** Determination of threshold values in healthy cows and those with endometritis (mild and moderate) using ROC analysis.

Variables	Groups	Cutoff	AUC*	Sensitivity (%)	Specificity (%)
Hp (µg/ml)	Healthy-ME	85	0.813	0.95	0.575
SAA (µg/ml)	Healthy-ME	16.25	0.85	0.95	0.55
	Healthy-E	19.275	0.993	0.95	0.67
Cp (mg/dl)	Healthy-ME	14.64	0.983	0.967	0.7
	Healthy-E	16.55	0.998	0.95	0.967

AUC=Area under the curve, Hp=Haptoglobin, SAA=Serum Amyloid A, Cp=Ceruloplasmin, ME=Mild endometritis, E=Moderate endometritis,

* AUC were different from AUC=0.5 in all variables, ROC=Receiver operator characteristic curve

### The levels of some biochemical parameters (AST, ALP, urea, creatinine, and TP) in the groups

Aspartate aminotransferase, ALP, urea, creatinine, and TP levels are presented in Table-2. In cows with endometritis, serum AST levels (ME=65.82±2.02, E=92.27±5.40, and SE=108.37±3.52 U/L) were significantly higher than in healthy cows (58.64±1.41 U/L; p=0.001). Similarly, serum ALP levels were higher in cows with endometritis than in healthy cows (38.69±1.77 U/L), and the serum ALP level increased as endometritis became more severe. The highest serum ALP levels were seen in cows with severe endometritis (68.52±3.49 U/L). Urea and creatinine levels were significantly higher in cows with endometritis than in healthy controls. TP levels in healthy cows and cows with mild and moderate endometritis were similar (p>0.05), whereas they were significantly higher in cows with severe endometritis than in healthy cows (p=0.01) (Table-2).

**Table-2:** The levels of some biochemical parameters in groups.

Variables	Healthy (n=30)	ME (n=30)	E (n=20)	SE (n=20)	p
AST (U/L)					
X̄±SEM	58.64^a^±1.41	65.82^a^±2.02	92.27^b^±5.40	108.37^c^±3.52	a:b: 0.001; a:c: 0.001; b:c: 0.005
ALP (U/L)					
X̄±SEM	38.69^a^±1.77	45.27^ab^±1.57	52.98^b^±1.69	68.52^c^±3.49	a:b:c: 0.001
Urea (mmol/L)					
X̄±SEM	7.62^a^±0.17	8.72^b^±0.28	9.04^bc^±0.33	9.24^bcd^±0.43	a:b: 0.02; a:bc: 0.06; a:bcd: 0.01
Creatinine (µmol/L)					
X̄±SEM	82.36^a^±2.07	93.62^b^±2.02	138.44^c^±4.14	144.42^cd^±5.13	a:b: 0.03; a:c: 0.001; a:cd: 0.001; b:c: 0.001
TP (g/L)					
X̄±SEM	67.77^a^±1.86	68.91^ac^±1.34	72.45^acb^±1.83	76.25^b^±2.45	a:b: 0.01; ac:b: 0.03

n=Number of cows, AST=Aspartate aminotransferase, ALP=Alkaline phosphatase, TP=Total protein, ME=Mild endometritis, E=Moderate endometritis, SE=Severe endometritis, Differences between average values are shown by different letters (a-c) on the same line (X̄= Mean value, SEM= Standard error of the mean, p= Value of statistical significance)

### Analysis of correlation between APPs and some biochemical parameters

The correlation between APPs and biochemical parameters is presented in Table-3. A significant correlation was found between serum ceruloplasmin levels and Hp (r=0.783, p=0.001) and SAA levels (r=0.739, p=0.001). A significant correlation was found between Hp, SAA, and ceruloplasmin levels and AST levels (r=0.642, r=0.700, and r=0.697, respectively) and ALP levels (r=0.640, r=0.615, and r=0.643, respectively) (p=0.001). Albumin levels correlated significantly only with serum AST level (r=0.205, p=0.04). When the correlation between biochemical parameters was evaluated, a significant correlation was found between serum AST, ALP, urea, and creatinine levels. No significant correlation was found between TP levels and albumin (r=0.031, p=0.76) and urea levels (r=0.061; p=0.54) (Table-3).

**Table-3:** Correlations between APPs and some biochemical parameters.

n=100	Hp	SAA	Cp	Alb	AST	ALP	Urea	Crea	TP
Hp	-	0.723*	0.783*	0.062	0.642*	0.640*	0.343*	0.658*	0.297^§^
SAA	0.723*	-	0.739*	0.157	0.700*	0.615*	0.323^&^	0.762*	0.277^§^
Cp	0.783*	0.739*	-	0.141	0.697*	0.643*	0.399*	0.721*	0.299^§^
Alb	0.062	0.157	0.141	-	0.205^§^	0.146	0.086	0.197	0.031
AST	0.642*	0.700*	0.697*	0.205^§^	-	0.585*	0.237*	0.692*	0.291^§^
ALP	0.640*	0.615*	0.643*	0.146	0.585*	-	0.285^§^	0.553*	0.209^§^
Urea	0.343*	0.323^&^	0.399*	0.086	0.237^§^	0.285^§^	-	0.334*	0.061
Crea	0.658*	0.762*	0.721*	0.197	0.692*	0.553*	0.334*	-	0.279^§^
TP	0.297^§^	0.277^§^	0.299^§^	0.031	0.291^§^	0.209^§^	0.061	0.279^§^	-

n=Number of cows, Statistical significance of correlations:

* p=0.001,

& p=0.01,

§ p<0.05 (Hp=Haptoglobin [µg/ml]; SAA=Serum Amyloid A (µg/ml), Cp=Ceruloplasmin (mg/dl), Alb=Albumin (g/dl), AST=Aspartate aminotransferase (U/L), ALP=Alkaline phosphatase (U/L), Urea (mmol/l), Crea=Creatinine (µmol/L), TP=Total protein (g/L). APP=Acute phase protein

### Serum APP levels were compared according to body condition score and parity

Cows were divided into two groups (BCS ≤2.75 and BCS >2.75) to determine the effects of BCS on APP levels. Hp, SAA, and ceruloplasmin levels in the group with BCS ≤2.75 (153.25±8.40 µg/ml, 23.23±1.05 µg/ml, and 19.69±0.67 mg/dl, respectively) were similar to the APP levels in the group with BCS >2.75 (148.21±10.78 µg/ml, 21.88±1.50 µg/ml, and 19.32±1.12 mg/dl, respectively), and no statistically significant difference was found (p>0.05). Hp (143.04±9.45 µg/ml), SAA (23.17±1.35 µg/ml), and ceruloplasmin (19.15±0.76 mg/dl) levels in primiparous cows were close to those in multiparous cows (160.09±9.07 µg/ml, 22.81±1.16 µg/ml, and 20.07±0.85 mg/dl, respectively), and no statistically significant difference was found (p>0.005) (Table-4).

**Table-4:** Comparison of APP levels by BCS and parity (X̄±SEM).

Variables	Groups	Haptoglobin (µg/ml)	Serum amyloid A (µg/ml)	Ceruloplasmin (mg/dl)
BCS	≤2.75 (n=72)	153.25±8.40	23.23±1.05	19.69±0.67
	>2.75 (n=28)	148.21±10.78	21.88±1.50	19.32±1.12
P		0.707	0.762	0.948
Parity	Primiparous (n=46)	143.04±9.45	23.17±1.35	19.15±0.76
	Multiparous (n=54)	160.09±9.07	22.81±1.16	20.07±0.85
P		0.969	0.280	0.442

n=Number of cows, BCS=Body condition score, =Mean value, SEM=Standard error of the mean; P=Value of statistical significance, APP=Acute phase protein

## Discussion

The aim of this study was to identify changes in serum ceruloplasmin levels in cows with endometritis based on the degree of severity and to establish whether or not serum ceruloplasmin levels can be used in the diagnosis of endometritis as an alternative to Hp and SAA levels. Many studies report that Hp, SAA, and APPs are reliable biomarkers in the diagnosis of uterine infections and facilitate monitoring of the treatment of endometritis [2,5,6,25-27]. Hp levels vary depending on the method of analysis, and the mean level reportedly ranges between 22 and 140 µg/ml in healthy animals [2,26,28,29]. Hp levels are reported to increase by 9 times if there is an infection [2,26,30]. SAA levels with a mean ranging between 16 and 22 µg/ml in healthy animals reportedly increase in the presence of uterine infections (35-99 µg/ml) [2,6,31]. The levels of albumin, a negative APP, in cows with endometritis (3.4±0.1 g/dl) were significantly lower than in healthy cows (3.6±0.1 g/dl) [7]. Musal *et al*. [32], on the other hand, reported that albumin levels were higher in cows with endometritis (3.34±0.12 g/dl) than in healthy cows (3.17±0.15 g/dl). This study found that Hp and SAA levels were significantly higher in cows with endometritis and that they increase as endometritis becomes more severe. On the other hand, albumin levels in the groups were similar, and the presence and severity of endometritis had no significant effect on albumin levels.

Another indicator used in the assessment of animal health and welfare is ceruloplasmin [33]. It is recognized as a valuable source of information, particularly for detecting mastitis in cows [34,35]. Ceruloplasmin levels were higher in the milk of cows with subclinical mastitis (3.35-8.02 U/g) than in the milk of healthy cows (0.72-2.11 U/g) [35]. The serum ceruloplasmin level in clinically healthy cows in the postpartum period (week 3-5) was reported to be 22.1±5.89 mg/dl [36]. Another study found this level to be 0.163±0.011 U/g in healthy cows [37]. Lamand and Levieux [38] reported that plasma ceruloplasmin levels were higher in sheep with uterine infections or inflammation than in healthy sheep. In reviewing the literature, we did not find a study analyzing changes in the ceruloplasmin levels of cows diagnosed with endometritis of varying degrees of severity. This study found that serum ceruloplasmin levels were significantly higher in cows with endometritis than in healthy cows (13.52±0.32 mg/dl) and that the ceruloplasmin level significantly increases as endometritis becomes more severe. Ceruloplasmin increases various enzyme (cytochrome C oxidase and Cu-Zn superoxide dismutase) activities together with copper, acts on immune system cells, and improves their phagocytosis and antimicrobial power. It also reduces the effects of oxygen radicals and protects cells against oxidative damage [11]. The increase in ceruloplasmin levels in cows with endometritis is thought to be caused by its effect on the immune system.

Body condition score, the most important indicator of the body’s energy balance, is one of the most important factors playing a role in the change in the amount of oxidative stress. This is, therefore, reported to cause changes in ceruloplasmin levels [39]. However, this study failed to find an effect of BCS on APP concentrations. When groups based on BCS were compared, the BCS of most of the cows in the group with BCS ≤2.75 was found to range between 2.50 and 2.75 and that of most of the cows in the group with BCS >2.75 were between 3 and 3.25. The fact that no statistically significant difference was found in APP levels is thought to be due to the fact that BCS was similar between the groups. There are also studies reporting that the number of parturitions changes APP levels. Metabolic changes arising from udder growth and development in primiparous cows after parturition are reportedly greater than in multiparous cows, and a higher amount of free oxygen radicals are generated. Ceruloplasmin levels are reported to be lower in primiparous cows than in multiparous cows because it is used as a buffering action against these radicals [39]. It has been reported that Hp levels are higher in primiparous cows in the week following parturition than in multiparous cows and that this is caused by the fact that damage to the uterus, vagina, and vulva may be more severe during parturition [8]. However, this study showed that the number of parturitions had no significant effect on APP levels. The fact that the number of parturitions does not change APP levels suggests that uterine infections cause similar stress conditions in primiparous and multiparous cows.

Changes in biochemical components are blamed for reproductive failures. Therefore, evaluation of the biochemical profile is important to eliminate the problems caused by endometritis. Both serum urea concentrations and TP levels are reported to be higher in cows with endometritis than in healthy cows [40]. However, there are also studies reporting that endometritis does not change serum urea concentrations [17]. This study found that urea concentrations are higher in cows with endometritis and that they increase as endometritis becomes more severe. The elevated urea concentrations are reported have the effect of changing uterine pH, weakening the local immune system, and increasing the severity of the infection [41]. This explains why the highest concentrations of urea are seen in the group with severe endometritis. A significant correlation is reported between urea concentrations and TP levels during the postpartum period (parturition-week 9 postpartum) [33]. Serum AST levels are reported to be higher in cows with endometritis than in healthy cows [17,42]. This study found that serum AST and ALP levels increase with endometritis, which is consistent with the aforementioned study. Tóthová *et al*. [33] found no correlation between urea concentrations and Hp, SAA, and albumin levels, but there was a significant correlation between creatinine levels and SAA, albumin, TP, and urea levels in a study they conducted. This study found significant correlations between all biochemical parameters except for TP both with each other and with APPs. As Burke *et al*. [17] reported that all these results show that endometritis impairs liver functions (as a result of toxins produced during infection indirectly reaching the liver).

## Conclusion

It was determined that endometritis changes both APP levels and biochemical parameters. Serum ceruloplasmin levels in cows with endometritis which were not known before were significantly higher in the presence of endometritis, and the severity of endometritis plays an important role in this increase. A significant correlation was found between serum ceruloplasmin levels and Hp and SAA levels. It was concluded that ceruloplasmin levels can be used in the diagnosis of endometritis as an alternative to Hp and SAA levels. Routine laboratory analyses of APPs can help reduce financial losses significantly by facilitating early diagnosis of diseases affecting reproduction, such as endometritis and monitoring treatment efficacy.

## Authors’ Contributions

This work was carried out in collaboration between all authors. SK, CK and AC: Designed the experimental procedures. SK and CK: Conducted the research work. OM: Helped in laboratory analysis. SK, AC, KB: Prepared figures, tables, revised and submitted the manuscript. All authors read and approved the final manuscript.
